# Reclassification of Adolescent Ambulatory Prehypertension and Unclassified Blood Pressures by 2022 American Heart Association Pediatric Ambulatory Blood Pressure Monitoring Guidelines

**DOI:** 10.21203/rs.3.rs-3074122/v1

**Published:** 2023-06-26

**Authors:** Taylor Hill-Horowitz, Kumail Merchant, Laura Castellanos Reyes, Pamela Singer, Haripriya Dukkipati, Rachel Frank, Christine B Sethna, Abby Basalely

**Affiliations:** Cohen Children’s Medical Center; NYU Langone Hospital - Long Island; Cohen Children’s Medical Center; Cohen Children’s Medical Center; Cohen Children’s Medical Center; Cohen Children’s Medical Center; Cohen Children’s Medical Center; Cohen Children’s Medical Center

**Keywords:** 2022 American Heart Association Pediatric Ambulatory Blood Pressure Monitoring Guidelines Update, Prehypertension, Blood Pressure Loads, Reclassification, Phenotype Switching, Repeat ABPMs

## Abstract

**Background:**

The 2022 American Heart Association (AHA) pediatric ambulatory blood pressure monitoring (ABPM) guidelines eliminated the prehypertension phenotype and blood pressure loads in ABPM interpretation criteria. Adolescents who were prehypertensive or unclassified according to the 2014 AHA pediatric ABPM guidelines will be reclassified as having hypertension or normotension. The epidemiology and association of reclassification phenotype with target organ damage (TOD) is not yet known.

**Methods:**

A single center retrospective review of adolescents ages 13–21 years old between 2015–2022 was performed. Adolescents diagnosed with prehypertension or unclassified by the 2014 AHA pediatric ABPM guidelines were reclassified by the 2022 definitions. Logistic regression models adjusted for body mass index z-score evaluated the association of reclassification phenotype with left ventricular hypertrophy (LVH).

**Results:**

Among 88 adolescents with prehypertension, 68% (N = 60) were reclassified as hypertensive. The majority (58%, N = 35) of hypertensive reclassification was based on isolated nocturnal blood pressures ≥ 110/65 mmHg. Taller males were more likely to reclassify as hypertensive. Adolescents reclassified as hypertensive had a greater-than-six-fold increased odds of LVH in adjusted models [OR 6.4 95%CI 1.2–33.0, p = 0.027]. Of 40 adolescents with unclassified blood pressures, 37.5% (N = 15) reclassified to normotension. There were no significant clinical or demographic variables associated with reclassification category nor was there an association with LVH.

**Conclusions:**

The new ABPM guidelines effectively reclassify adolescents who were previously prehypertensive as normotensive or hypertensive based on risk of TOD. Further studies are needed to describe the long-term outcomes of ABPM phenotypes with the implementation of these guidelines.

## Introduction

In 2022, the American Heart Association (AHA) published updated guidelines for pediatric ambulatory blood pressure monitoring (ABPM) interpretation. Both the classification of prehypertension and blood pressure loads were eliminated, and a lower threshold to diagnose pediatric hypertension was implemented. These updates align pediatric ABPM interpretation guidelines with those of adults and are in congruence with recent research which has shown no association between blood pressure loads and target organ damage (TOD) [1].

Prehypertension was first described in the Seventh Report of the Joint National Committee on Prevention, Detection, Evaluation, and Treatment of High Blood Pressure [2]. According to the 2014 AHA pediatric ABPM guidelines, prehypertension was defined as elevated clinic blood pressures, normal ambulatory blood pressures, and blood pressures above the 95th percentile for at least a quarter of the time during ambulatory monitoring (Supplementary Table 1) [3]. It was not intended to be a disease category but, rather, a way of identifying children at risk of hypertension. The Seventh Report recommended preventative lifestyle modification, such as diets rich in fruits and vegetables and at least 30 minutes of exercise per day, and annual surveillance by ABPM for prehypertensive children. Antihypertensive medications were recommended for consideration in children with comorbidities, such as diabetes or kidney disease [2].

A major issue surrounding the phenotype, as Flynn et al. raised in the 2014 AHA pediatric ABPM guidelines, was that prehypertension had no correlative phenotype in adult ABPM interpretation [3]. Additionally, prior to 2022, the blood pressure thresholds for hypertension differed between adult and pediatric guidelines. Since 2017, adult ambulatory hypertension guidelines used cutoffs of mean 24h systolic blood pressure ≥ 125 mmHg or diastolic blood pressure ≥ 75 mmHg, mean wake systolic blood pressure ≥ 130 mmHg or diastolic blood pressure ≥ 80 mmHg, and mean sleep systolic blood pressure ≥ 110 or diastolic blood pressure ≥ 65 mmHg [4]. Alternatively, pediatric diagnoses of hypertension used the threshold of blood pressures above the 95th percentile for age and sex [3]. For many adolescent patients, particularly tall males, the 95th percentile blood pressure thresholds surpassed adult cutoffs. Thus, transitioning adolescent patients to adult care was complicated, as a patient would be evaluated by different criteria when they turned 18 and often reclassify as hypertensive [5]. Emerging data from the Study of High Blood Pressure in Pediatrics: Adult Hypertension Onset in Youth (SHIP-AHOY) have shown that adult ambulatory cutoffs have increased sensitivity and specificity to detect left ventricular hypertrophy (LVH) in adolescents as compared to pediatric thresholds of the 95th percentile blood pressures [6]. The incongruence of adult blood pressure thresholds and hypertensive phenotypes with pediatric ABPM interpretation schema prompted the updated AHA guidelines to eliminate the phenotype of prehypertension from ABPM diagnostic criteria and lower the threshold for a diagnosis of pediatric hypertension to align with adult definitions [7, 8].

The third major change by the 2022 AHA pediatric ABPM guidelines was the removal of blood pressure loads from ABPM interpretation. The three-pronged blood pressure classification system, using ambulatory and clinic blood pressures in conjunction with blood pressure loads, left an estimated 20–40% of patients “unclassified,” as they did not align with any phenotype of the 2014 AHA pediatric ABPM guidelines [6]. In 2020, Lee et al. studied 533 children from the Chronic Kidney Disease in Children (CKiD) cohort and found no meaningful association between blood pressure loads and chronic kidney disease (CKD) progression or cardiovascular outcomes [9]. The SHIP-AHOY study corroborated these results in children without CKD [6]. The 2022 AHA pediatric ABPM guidelines have simplified diagnostic criteria by removing blood pressure loads, ensuring that every patient will fit into one of four blood pressure phenotypes: normotension, white coat hypertension (WCH), masked hypertension, and ambulatory hypertension.

As the 2022 guidelines have implemented lower thresholds for hypertension, eliminated the category of prehypertension, and provided an ABPM phenotype regardless of blood pressure load, prehypertensive and unclassified adolescents will reclassify as either normotensive or hypertensive. Data are limited regarding the epidemiology of reclassification, as well as the association of new ABPM phenotype with TOD. Therefore, this study aimed to (1) describe the epidemiology of reclassification of adolescents with prehypertension; (2) describe the epidemiology of reclassification for adolescents who were unclassified by the 2014 AHA pediatric ABPM guidelines; and (3) evaluate the association of new diagnostic category with LVH.

## Methods

A retrospective review was conducted of ABPM reports from adolescents between the ages of 13 and 21 years old seen at Cohen Children’s Medical Center from 2015 to 2022. ABPM reports were included in the analysis if they had at least: 40 total blood pressure readings, 1 blood pressure measurement per hour, and 65% successful blood pressure readings. Previous diagnosis of hypertension, severity of hypertension, CKD status, and transplant status were not criteria for exclusion from analysis [10]. Clinic blood pressures measured within two weeks prior to ABPM placement were extracted from the electronic medical record. The study was approved by the Institutional Review Board of Northwell Health.

Spacelabs ABPM devices (model 90217A; Spacelabs Healthcare) were utilized. Participants were instructed to wear the ABPM for 24 hours on their nondominant arm. Sleep and wake timepoints were determined by patient report. ABPM reports of each participant were interpreted by both the 2014 and 2022 AHA pediatric ABPM guidelines’ criteria [1, 3].

Echocardiography reports were obtained from the medical record. Only echocardiograms performed within 6 months of the ABPM were analyzed. Left ventricular mass index (LVMI) was calculated for each echocardiogram utilizing M-mode measurements for interventricular septum, left ventricular internal diameter, and left ventricular posterior wall and the Devereux formula for LVMI by height [4, 11–13]. LVH was defined as LVMI > 95th percentile for age and sex [11, 14].

Prehypertension was defined according to the 2014 AHA pediatric ABPM guidelines as clinic systolic or diastolic blood pressures ≥ 90th percentile or > 120/80 mmHg (whichever is lower), ambulatory systolic or diastolic blood pressures < 95th percentile, and blood pressure loads ≥ 25% (Supplementary Table 1). Unclassified ABPM was defined as blood pressures that did not meet criteria for a diagnostic category according to the 2014 schema, which manifested as either 1) clinic blood pressure < 90th percentile with ambulatory blood pressures < 95th percentile and loads ≥ 25% or 2) clinic blood pressure > 90th and < 95th percentile with loads < 25% (Supplementary Table 1). The 2014 AHA pediatric ABPM interpretation schema utilized normative reference data from Wuhl et al. [11].

Primary exposures were ABPM reclassification phenotype of hypertension or normotension. Hypertension included diagnoses of masked or ambulatory hypertension. Normotension included those who were diagnosed as WCH as well as normotension, as ambulatory blood pressures for WCH are in the normal range (Supplementary Table 1). The primary outcome was LVH.

Participant demographic information was obtained from the electronic medical record. Body mass index (BMI) was calculated by height and weight and converted to z-scores.

Demographic and clinical characteristics were expressed as median/interquartile range (IQR) and percentages as appropriate. Pearson’s chi-squared and Wilcoxon rank-sum tests compared adolescents’ reclassified ABPM phenotypes. Logistic regression models were used to evaluate the association of ABPM phenotype and LVH. Models were adjusted for BMI z-score percentile, as age and sex are colinear with the outcome of LVH as defined by the 95th percentile LVMI for age and sex. P-values ≤ 0.05 were considered statistically significant for all analyses. STATA version 16 (StataCorp LLC) statistical packages were used for analysis.

## Results

The cohort consisted of 353 ABPMs from 334 unique adolescents who met inclusion criteria.

### Prehypertension

**Figure 1.**
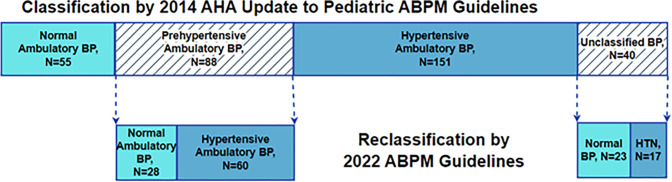
Overall Distribution of Cohort by the 2014 Guidelines and Reclassification of Prehypertension and Unclassified BPs by the 2022 AHA Pediatric ABPM Guidelines. Blood pressures (BPs) from adolescents ages 13 to 21 were first classified into BP phenotypes using the 2014 American Heart Association (AHA) Update to Pediatric Ambulatory Blood Pressure Monitoring (ABPM) Guidelines. Those who classified as prehypertensive or were left unclassified were then categorized by the BP phenotype definitions described in the 2022 AHA pediatric ABPM guidelines. 68% of adolescents with prehypertension reclassified to hypertension (HTN). 43% of adolescents with unclassified BPs reclassified to HTN.

Of 334 adolescents, 88 (26%) were diagnosed with prehypertension according to the 2014 AHA pediatric ABPM guidelines. Adolescents with prehypertension were predominantly male with a median age of 16 years (IQR, 14–17 years), height of 170 cm (IQR, 164–178 cm), and BMI z-score percentile of 0.95 (IQR, 0.81–0.99). The 2022 AHA pediatric ABPM guidelines led to the reclassification of 68% of these adolescents as hypertensive (Fig. 1). Males comprised a larger percentage of hypertension as compared to normotension (87% vs 57%, p = 0.010), and reclassification to hypertension was associated with a taller median height (172 cm vs 168 cm, p = 0.046) (Table 1). Systolic blood pressures were significantly higher for those reclassified to hypertension (p < 0.001), and 25% (N = 15) of those who reclassified to hypertension met the criteria by elevated systolic blood pressures alone. There was no significant difference in age, BMI z-score percentile, or diastolic blood pressure among reclassified individuals (Table 1).

Distribution of specific reclassification phenotypes were as follows: 7% (N = 6) to normotension, 25% (N = 22) to WCH, 17% (N = 15) to masked hypertension, and 51% (N = 45) to hypertension. Elevated nocturnal blood pressures were the sole reason for reclassification to hypertension in 58% (N = 35/60) of the newly hypertensive cohort (Supplementary Table 2).

Echocardiograms were available for 63% (N = 55) of adolescents with prehypertension, and 38% (N = 21) demonstrated LVH. Thus, prehypertension was not significantly associated with LVH. Only 13% (N = 2) of those who were reclassified to normotension demonstrated LVH, compared to 46% (N = 19) of those reclassified to hypertension. In contrast to the phenotype of prehypertension, reclassification to hypertension had a greater-than-6-fold increase in LVH compared to reclassification to normotension in models adjusted for BMI z-score percentile [OR 6.4 95%CI 1.2–33.0] (p = 0.027). Adolescents with either isolated daytime hypertension or day-night sustained hypertension demonstrated a 65% prevalence of LVH (N = 11/17, p = 0.049). The prevalence of LVH was lower among those with prehypertension who reclassified with isolated nocturnal hypertension (33%, N = 8/24) (Supplementary Table 3).

**Figure 2.**
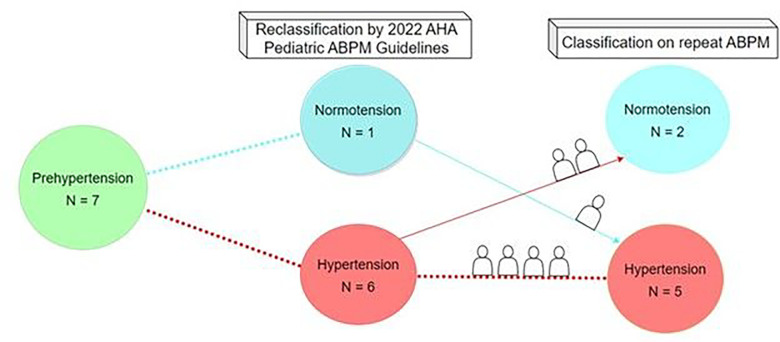
Change in Phenotype of Adolescents Reclassified from Prehypertension on Repeat ABPM Performed within 4 years. 7 adolescents with prehypertension according to the 2014 American Heart Association (AHA) Update to Pediatric Ambulatory Blood Pressure Monitoring (ABPM) Guidelines had repeat ABPMs performed within 4 years. The initial and subsequent ABPMs were reclassified by the 2022 AHA pediatric ABPM guidelines, and phenotype switching within 4 years was as follows: 2/6 adolescents with hypertension became normotensive, and 1/1 adolescents with normotension became hypertensive.

Seven adolescents had repeat ABPMs within 4 years of their diagnosis of prehypertension. According to the 2022 AHA pediatric ABPM guidelines, 6 reclassified to hypertension. One reclassified to normotension yet developed hypertension upon follow-up ABPM (Fig. 2).

### Unclassified ABPMs

Using the 2014 ABPM guidelines, 12% (N = 40) of adolescents were unclassified. This cohort was predominantly male with a median age of 16 years (IQR, 15–17 years), height of 170.5 cm (IQR, 165–180 cm), and BMI z-score percentile of 0.91 (IQR, 0.82–0.97). Among this group, 42.5% (N = 17) reclassified to hypertension, and 57.5% (N = 23) reclassified to normotension (Fig. 1). Ambulatory blood pressures were higher for adolescents who reclassified as hypertensive than normotensive (p < 0.01) (Table 2). There was no association between reclassification phenotype and age, sex, height, or BMI z-score percentile. Echocardiograms were available for 63% (N = 25/40) of the unclassified sample. Reclassification phenotype for this sample was not significantly associated with LVH [OR 3.3 95%CI 0.6–18.5]. The distribution of reclassification was 37.5% (N = 15) to normotension, 20% (N = 8) to WCH, 35% (N = 14) to masked hypertension, and 7.5% (N = 3) to ambulatory hypertension.

## Discussion

This cohort is the largest to date to study the reclassification of adolescents with prehypertension by the 2022 AHA pediatric ABPM guidelines. As hypothesized, the majority (68%) of prehypertensive patients reclassified as hypertensive, and taller males demonstrated higher rates of hypertension. Interestingly, over half (58%) of adolescents reclassified from prehypertension to hypertension based on isolated nocturnal hypertension, and of this cohort, 1/3 demonstrated LVH. While models of prehypertension were not associated with LVH, hypertensive reclassification according to the 2022 guidelines was associated with substantially increased odds of LVH. This study supports the 2022 AHA pediatric ABPM guidelines’ reclassification of prehypertensive adolescents to hypertensive or normotensive phenotypes as it better discriminates risk of TOD. While the new guidelines successfully categorized adolescents with previously unclassified blood pressures, no significant association was appreciated between reclassification phenotype and LVH.

The rate of hypertensive reclassification among prehypertensive adolescents in this study was similar to a recent study by Ahlenius et al. of children ages 5 to 20 years old from the University of Texas Health Sciences Center at San Antonio, which found that 70% (N = 46/66) of children with prehypertension reclassified as hypertensive according to the 2022 AHA pediatric ABPM guidelines. Ahlenius’ cohort was smaller and younger than our cohort, and the majority of their cohort in Texas identified as Hispanic. In contrast to our results, Ahlenius et al. did not find height and sex to be significantly associated with reclassification phenotype, though their results did suggest that reclassification to normotension was more common among shorter females.

The discrepancy may be explained by the limited sample size and/or younger age of their cohort from the University of Texas Health Sciences Center. This study’s concentration on prehypertensive adolescents with a median age of 16 (IQR, 14–17) may have driven the significant association of height and sex with reclassification phenotype. The 95th percentile blood pressures for tall adolescent males are greater than adult thresholds, making these adolescents the most likely to reclassify to hypertension [8]. For example, a 175 cm, 17-year-old boy with an average wake systolic blood pressure of 135 mmHg was not hypertensive according to the 2014 AHA pediatric ABPM guidelines. However, according to the 2022 definitions of ABPM phenotypes, he would be diagnosed with ambulatory hypertension [8]. Therefore, adolescent cohorts may be more likely to demonstrate the significant association of height and sex with reclassification phenotype by the 2022 AHA pediatric ABPM guidelines.

An important finding from this study is that most adolescents with prehypertension reclassified based on sleep blood pressures only. Thus, this cohort brings attention to the lowered threshold of nocturnal hypertension (110/65 mmHg), which has been an ongoing area of interest in the practice of pediatric nephrology. Data on pediatric isolated nocturnal hypertension thus far is limited to studies utilizing 95th percentile blood pressure cutoffs. In analyses of the CKiD cohort using 95th percentile thresholds, isolated nocturnal hypertension was associated with low birthweight, older age, and taller height and was appreciated earlier in the course of CKD. TOD, measured by carotid intima-media thickness (cIMT) and pulse wave velocity, was significantly increased among children and adolescents with isolated nocturnal hypertension as compared to normotension [9].

Existing research is inconclusive as to whether isolated nocturnal hypertension is associated with worse cardiovascular outcomes than isolated daytime hypertension. Right ventricular mechanics are shown to be worse in adults with isolated nocturnal hypertension than isolated daytime hypertension [2]. These same trends were not found in children: a study of 102 hypertensive ABPMs showed that children with isolated nocturnal hypertension had significantly higher LVMI when adjusted for age than normotensive children, but LVMIs of those with isolated nighttime and isolated daytime hypertension were similar [15]. In our cohort, one third of adolescents with isolated nocturnal hypertension demonstrated LVH, though the prevalence of LVH was lower in isolated nocturnal hypertension as compared to isolated daytime or sustained day-night hypertension.

The first study to evaluate pediatric nocturnal hypertension in terms of static thresholds occurred in 2021 with the SHIP-AHOY cohort. While they did not specifically evaluate isolated nocturnal hypertension, Hamdani et al. found a threshold for nocturnal hypertension of 110/65 mmHg to be the optimal sensitivity-specificity balance to identify pediatric LVH in comparison to 95th percentile cutoffs or the 120/80 mmHg threshold used by the European Society of Hypertension [6]. Our study is the first to demonstrate the prevalence of isolated nocturnal hypertension, and its association with LVH, among those who reclassified from prehypertension to hypertension.

Although the original goal of the prehypertension phenotype was to identify those at highest risk of developing hypertension, the 2014 AHA pediatric ABPM guidelines also reported that children with prehypertension had increased odds of TOD [3]. Eliminating this phenotype raises the question of whether prehypertensive children who reclassify as normotensive will have appropriate clinical monitoring to identify the potential development of hypertension.

Progression from prehypertension to hypertension was studied in a 2008 analysis of the National Childhood Blood Pressure database, investigating longitudinal blood pressure phenotype switching. Of the pediatric patients diagnosed with prehypertension, 14% of males (N = 128/882) and 12% of females (N = 71/588) had developed hypertension within two years [16]. Similarly, Hanevold and colleagues analyzed 124 children in Seattle and Pittsburgh who were referred for elevated clinic blood pressures with repeat ABPM at least six months apart, and they found annual rates of progression from prehypertension to hypertension to be 19% [17].

Our results suggest that the 2022 ABPM interpretation guidelines have successfully simplified ABPM interpretation criteria without “missing” patients who should receive recommendations for increased monitoring. Reconciling existing data with our results, it is imperative to recognize that past studies on ABPM phenotype-switching utilized the 2014 definition of hypertension with higher thresholds [16, 17]. With the implementation of lower thresholds for pediatric hypertension by the 2022 AHA pediatric ABPM guidelines, the patients with prehypertension at highest risk of developing hypertension will likely have been reclassified as hypertensive, recommending them for regular ABPM surveillance. The increased sensitivity of the 2022 definition of hypertension suggests a low likelihood of underestimating ABPM phenotype progression and TOD in patients who reclassify as normotensive. Of note, the one prehypertensive patient who reclassified as normotensive did develop hypertension within 4 years, maintaining the importance of clinic blood pressure surveillance at routine visits in all adolescents.

Prehypertension was not associated with LVH, but reclassification to hypertension conferred a greater-than-6-fold increase in odds of LVH as compared to normotensive reclassification. This supports the 2022 AHA pediatric ABPM guidelines’ removal of prehypertension, as the updated ABPM phenotypes effectively discriminate among adolescents at-risk of TOD. However, the lack of predictive value of prehypertension found in this cohort was at odds with previous research. A 2020 study by Obrycki et al. of 304 adolescents found that stroke volume and cardiac output were greater and cIMT Z-scores were higher in adolescents with prehypertension as compared to those with normotension but were similar between prehypertension and other hypertensive phenotypes [18]. Stabouli et al. studied a cohort of 124 children, ages 5 to 18, and found that both prehypertension and hypertension had a significantly higher prevalence of LVH as compared to normotension (p < 0.05) [19]. Differences in the prevalence of LVH among prehypertension may be attributed to Stabouli et al.’s younger cohort or Obrycki et al.’s more generous definition of LVH as LVMI ≥ 38.6 g/m^2.7^, compared to this study’s definition of LVH as LVMI > 95th percentile. The lowered thresholds for hypertension implemented with the 2022 AHA pediatric ABPM guidelines reclassified many prehypertensive adolescents who demonstrated LVH as hypertensive.

Most adolescents who were previously unclassified based on their clinic and ambulatory blood pressures and loads reclassified by the 2022 AHA pediatric ABPM guidelines to normotension (37.5%) or masked hypertension (35%). As over one-third of the unclassified cohort was normotensive on clinic blood pressures yet exhibited hypertension on 24-hour monitoring, these conclusions emphasize the importance of ABPMs to corroborate clinic blood pressures and of annual surveillance on at-risk patients. This study supports the 2022 AHA pediatric ABPM guidelines’ removal of loads from ABPM interpretation as it assigns a diagnostic category to everyone, thus standardizing follow-up and surveillance. As hypothesized, both systolic and diastolic blood pressures were significantly higher in reclassification to hypertension. While this study did not find an association of reclassification phenotype with presence of LVH, this finding should be interpreted with caution as it may have been due to the limited sample size.

This was a single center study, limiting the predictive value of our results. Echocardiograph reports were only available for 63% of our population of interest: 55 out of 88 prehypertensive adolescents and 25 out of 40 unclassified adolescents. Additional information, such as indication for ABPM and prevalence comorbidities in the patient population, would have helped us better understand our cohort but were not available for analyses.

Nonetheless, this is the largest study to date to evaluate reclassification by the 2022 AHA pediatric ABPM guidelines. The outcomes are clearly defined, as ambulatory blood pressures were used in conjunction with clinic blood pressures to define phenotypes by both the 2014 and 2022 guidelines. Though race was not collected in this sample, Cohen Children’s Medical Center serves a diverse population in Queens, NY. In conclusion, the 2022 AHA pediatric ABPM guidelines better discriminate between adolescents at risk of TOD. Most prehypertensive adolescents reclassified to hypertension, specifically the masked hypertension phenotype due to elevated nocturnal blood pressures. This is reflective of the considerable increase in sensitivity of the nocturnal hypertension thresholds implemented with the 2022 ABPM phenotype definitions, which may lead to a disproportionate increase in the rate of tall, adolescent males diagnosed with hypertension. All unclassified adolescents were assigned a diagnostic phenotype with the implementation of the 2022 guidelines, supporting the simplification of ABPM interpretation criteria through the removal of blood pressure loads. Prospective studies in larger cohorts are needed to better describe cardiovascular outcomes in adolescents with hypertension. Additionally, future cost-effectiveness analyses should examine the additional screening that will follow the increased prevalence of hypertension, which has more comprehensive follow-up recommendations than prehypertension. Analysis of sequential ABPMs to evaluate phenotype-switching according to the 2022 AHA guidelines will be important for prognosis and counseling of patients.

## Figures and Tables

**Table 1:** Demographics and Cardiovascular Outcomes after Reclassification of Adolescents with Prehypertension

Reclassification of Prehypertension by the 2022 AHA Pediatric ABPM Guidelines			
Variables*	Normotensive Ambulatory Blood Pressure‡(n = 28)	Hypertensive Ambulatory Blood Pressure‡(n = 60)	P-Value
Median age - *years*	16 (14.5–17)	15 (14–17)	0.73
Male sex (n%)	12 (57%)	46 (87%)	0.010
Median height - *cm*	168 (160–177)	172 (168–179)	0.046
Median BMI	29.4 (22.4–35.0)	26.7 (23.4–31.0)	0.39
BMI Z-score percentile	98 (65–99)	94 (83–98)	0.34
Median 24h systolic blood pressure - *mmHg*	115 (112–120)	123 (119.5–127)	< 0.001
Median 24h diastolic blood pressure - *mmHg*	67 (66–71)	69 (66–72.5)	0.23
Median wake systolic blood pressure - *mmHg*	121 (116–127)	126 (121.5–132)	< 0.001
Median wake diastolic blood pressure - *mmHg*	72 (69–77)	72 (67–76)	0.97
Median sleep systolic blood pressure - *mmHg*	106 (104–108)	113 (111–117)	< 0.001
Median sleep diastolic blood pressure - *mmHg*	60 (57–63)	61 (58–63)	0.27
LVMI > 95th percentile (n%)	2 (13%)	19 (46%)	0.024
LVMI > 51 m/g^2.7^ (n%)	0 (0%)	6 (15%)	0.12

* All continuous variable represented as median (IQR)

‡ Reclassified ambulatory blood pressures based on 2022 AHA pediatric ABPM guidelines: Hypertension refers to masked or sustained hypertension, and normotension includes white coat hypertension

**Table 2:** Demographics and Outcomes after 2022 Reclassification of Adolescents Previously Unclassified by 2014 American Heart Association Pediatric Ambulatory Blood Pressure Monitoring Guidelines

Demographics and Clinical Variables of Unclassified Adolescents by Reclassification Phenotype*			
Variables^†^	Normotensive Ambulatory Blood Pressure‡(n = 23)	Hypertensive Ambulatory Blood Pressure‡(n = 17)	P-Value
Age - *years*	16 (15–17)	16 (15–17)	0.73
Male sex (n%)	16 (84)	13 (81)	1.00
Height - *cm*	171 (164–178)	170 (166–184)	0.43
BMI	25 (24–34)	26 (24–29)	0.63
BMI Z-score percentile	91 (80–99)	91 (85–96)	0.79
Median 24h systolic blood pressure - *mmHg*	112 (107–119)	125 (119–126)	< 0.001
Median 24h diastolic blood pressure - *mmHg*	62 (59–65)	69 (64–71)	0.009
Median wake systolic blood pressure - *mmHg*	113 (109–123)	128 (119–132)	< 0.001
Median wake diastolic blood pressure - *mmHg*	66 (61–70)	72 (68–76)	0.009
Median sleep systolic blood pressure - *mmHg*	103 (97–106)	116 (112–119)	< 0.001
Median sleep diastolic blood pressure - *mmHg*	53 (51–57)	60 (57–62)	< 0.001
LVMI > 95th percentile (n%)	3 (23%)	6 (50%)	0.16
LVMI > 51 (n%)	0 (0%)	1 (8%)	0.29

* Unclassified defined as blood pressures that did not fit a diagnostic category according to the 2014 definitions of ABPM phenotypes, either 1) clinic blood pressure < 90th percentile with ambulatory blood pressures < 95th percentile and loads ≥ 25% or 2) clinic blood pressure > 90th and < 95th percentile with loads < 25%

† All continuous variable represented as median (IQR)

‡ Reclassified ambulatory blood pressures based on 2022 AHA pediatric ABPM guidelines: Hypertension refers to masked or sustained hypertension, and normotension includes white coat hypertension
